# *In vitro* activity of fosfomycin against *Campylobacter* isolates from poultry and wild birds

**DOI:** 10.1371/journal.pone.0200853

**Published:** 2018-07-17

**Authors:** Bai Wei, Min Kang

**Affiliations:** Department of Veterinary Infectious Diseases and Avian Diseases, College of Veterinary Medicine and Center for Poultry Diseases Control, Chonbuk National University, Iksan, South Korea; Northeastern University, UNITED STATES

## Abstract

Fosfomycin is a broad-spectrum antibiotic with the activity against both Gram-positive and Gram-negative pathogens by inhibiting the bacterial cell wall synthesis. Given the potential therapeutic efficacy of fosfomycin against *Campylobacter* spp., the aim of the present study was to determine the *in vitro* fosfomycin susceptibility of *Campylobacter* isolates from avian sources including poultry and wild birds. A total of eight (1.8%) strains of *Campylobacter* including five *C*. *jejuni* strains isolated from ducks and three *C*. *coli* strains isolated from chickens and duck showed resistance to fosfomycin, with MICs ranging from 64 to ≥ 256 μg/mL. The extent of fosfomycin resistance was 0%, 0.9% and 3.9% in wild birds, chicken and ducks respectively. The MIC_50,_ MIC_90_, and MIC_100_ values were 8, 32, and 32 μg/mL respectively in wild bird, 32, 32, and 64 μg/mL respectively in chicken, and 32, 32, and ≥ 256 μg/mL respectively in ducks. All eight fosfomycin-resistant *Campylobacter* strains were multidrug resistant; six were also resistant to fluoroquinolones, ampicillin, and tetracycline, and two were also resistant to fluoroquinolones, ampicillin, tetracycline, and macrolides. However, the fosfomycin resistance gene *fosX*^*CC*^ was not detected in the eight fosfomycin-resistant strains. Because food animals can harbor fosfomycin-resistant *Campylobacter* and transmit them to humans, greater efforts are needed to monitor the prevalence of fosfomycin resistance in *Campylobacter* strains isolated from such animals.

## Introduction

*Campylobacter jejuni* and *C*. *coli* are recognized as major causes of human gastroenteritis worldwide. Campylobacteriosis is usually a self-limiting infection, and antimicrobial therapy is required only in cases of severe, prolonged, or systemic infections, or to control infection in high-risk groups [1]. In such cases, macrolides and fluoroquinolones are the drugs of choice for treatment. Unfortunately, high ciprofloxacin resistance and extremely high resistance had been found in European and Asian countries, respectively; 54.6% human isolates was with ciprofloxacin resistance in Europe, 86.7% in China and 95.2% in Korea [2–4]. The incidence of macrolide resistance was found to be lower than that of fluoroquinolone resistance in *Campylobacter* spp. However, increased resistance to macrolides in *Campylobacter* spp., and high macrolides resistance in *C*. *coli* has also been reported [2]. In addition, most of the macrolide-resistant *Campylobacter* strains were also found to be resistant to fluoroquinolones, 94.7% of erythromycin-resistant *Campylobacter* strains isolated from humans were also resistant to ciprofloxacin in Finland and all erythromycin-resistant strains co-resistant to ciprofloxacin in Korea [4, 5]. Moreover, the recent increase in incidence of multidrug resistant *Campylobacter* infections has increased treatment complexity, and few options exist for treatment of severe *Campylobacter* infection [2, 6]. Considering the significant risks of longer disease duration, increased morbidity, and higher costs that patients infected with drug-resistant *Campylobacter* may have [7], new antibiotics are urgently needed [8]. The discovery of a highly drug-resistant *Campylobacter* strain (resistant to fluoroquinolones, macrolides, beta-lactams, aminoglycosides, phenicols, lincosamides, streptomycin, and tetracyclines) in an international traveler with diarrhea enteritis [9] highlights the urgent necessity to find new antimicrobial agents to treat such *Campylobacter* infections and address the risk of treatment failure [10].

Despite pharmaceutical research and development focusing on new antimicrobial agents against *Campylobacter* including first-line antibiotic-resistant strains, the efforts have been limited to comparison with antibiotics active against other *Enterobacteriaceae* members [11]. Several attempts have also been made to develop therapeutic strategies against *Campylobacter* including use of selective alternative antibiotics, efflux pump inhibitors combined with antibiotics, antimicrobial peptides, and plant extracts, most of which have failed to meet the clinical requirements which the conventional antibiotics fulfill [7, 12–14]. Fosfomycin, which inhibits bacterial cell wall synthesis and shows activity against most multidrug-resistant bacteria in *Enterobacteriaceae*, is one of the antibiotics used clinically to treat *Campylobacter* infections [7, 15]. Moreover, fosfomycin shows high efficacy in inhibiting *Campylobacter* isolates *in vitro* as well as in treating *Campylobacter* enteritis in the clinical setting [7, 15, 16]. Although only a few studies have reported susceptibility of clinical isolates of *Campylobacter* spp. to fosfomycin, fosfomycin-resistant *Campylobacter* have been reported in humans with an increasing trend in the extent of resistance. This increase is evident on comparing the results of the following two Japanese studies. In 1984, the minimum inhibitory concentration (MIC) of fosfomycin against clinically isolated *C*. *jejuni* samples was consistently reported to be ≤ 12.5 μg/mL [15]; however, in 2008, the MIC_90_ was reported to have increased to 64 μg/mL [16]. There has been no extensive investigation of the susceptibility of *Campylobacter* strains isolated from food animals to fosfomycin, with most studies using a limited number of isolates or a limited number of animal sources. Sackey et al., [17] showed no fosfomycin resistance among 14 *Campylobacter* isolates from chicken. However, over 10% of *Campylobacter* spp. isolated from egg-laying hens showed resistance to fosfomycin (MIC > 32 μg/mL) [18]. Recently, a strain showing a high level (MIC > 512 μg/mL) of resistance to fosfomycin was isolated from swine [19]. Further, the presence of macrolide and fluoroquinolone resistance along with fosfomycin resistance among *Campylobacter* species found in humans and food animals is of great concern [19, 20].

*Campylobacter* spp. is widespread in nature with its principal reservoirs being the alimentary tract of wild and domesticated animals. Based on the optimal temperature required for *Campylobacter* growth, which is similar to that found naturally in avian intestines, poultry and wild birds have been suggest as natural reservoirs for *Campylobacter*. Among sporadic human cases, contact with live poultry and consumption of poultry meat have been identified as the major sources of infections [2]. Consequently, fosfomycin-resistant *Campylobacter* could be transmitted to humans from poultry in a direct or indirect manner. Because of the lack of fosfomycin resistance in field isolates of *Campylobacter* spp. and those from poultry in particular, understanding the level of fosfomycin resistance in *Campylobacter* found in poultry is important. Wild animals are not expected to be routinely exposed to antimicrobial agents, and occurrence of antibiotic resistance and identification of resistant *Campylobacter* spp. from wild animals would reflect primitive pre-existing resistance to antimicrobial agents [21]. The aim of the present study, therefore, was to determine the *in vitro* fosfomycin susceptibility of *Campylobacter* isolates from avian sources including poultry and wild birds. We also investigated the fosfomycin resistance mechanisms and clonal correlation among the resistant strains.

## Materials and methods

### Bacterial isolates

A total of 442 strains of *Campylobacter* (322 strains of *C*. *jejuni* and 120 strains *C*. *coli*) isolated from chickens, ducks, and wild birds from 2012 to 2016 were used in this study (Table 1). The isolation and PCR identification of these *Campylobacter* strains selected in this study has been previously described, 1) 155 strains of *Campylobacter* from the ducks (including broiler ducks and duck meat), 2) 227 strains from broiler chickens, breeder chickens, and chicken meat, and 3) 60 strains from the wild birds [22–24]. All wild birds are migratory birds and were collected from the migratory bird habitats along the migratory pathway. The sixty *Campylobacter* strains were recovered from 12 bird species including 7 families: 50 strains recovered from Anatidae (22 strains from Mandarin duck, 17 from Mallard, 7 from Spot-billed duck, 2 from Common teal, 1 from European wigeon and Greater white-fronted goose), 5 strains from Laridae (Black-tailed gull), and 1 strain each from Scolopacidae (Dunlin), Charadriidae (Greater sand plover), Corvidae (Azure-winged magpie), Sturnidae (White-cheeked starling) and Passeridae (Eurasian tree sparrow).

**Table 1:** MIC distribution of fosfomycin for *Campylobacter jejuni* and *C*. *coli* isolates from different sources.

Species	Source	Isolate No.	MIC (μg/mL)														Resistance No. (%)
			≤0.25	0.5	1	2	4	8	16	32	64	128	≥256	MIC_50_	MIC_90_	MIC_100_	
***C*. *jejuni***	Total	322	42			4	2	12	90	167	2	2	1	32	32	≥ 256	5 (1.6)
	Chickens	142	11			2		5	42	82				32	32	32	0
	Ducks	127	7			2		5	34	74	2	2	1	32	32	≥ 256	5 (3.9)
	Wild birds	53	24				2	2	14	11				8	32	32	0
***C*. *coli***	Total	120	8			1	1	9	32	66	2	1		32	32	128	3 (2.5)
	Chickens	85	6				1	7	18	51	2			32	32	64	2 (2.4)
	Ducks	28				1		1	13	12		1		16	32	128	1 (3.6)
	Wild birds	7	2					1	1	3				16	32	32	0
***Campylobacter***	Total	442	50			5	3	21	122	233	4	3	1	32	32	≥ 256	8 (1.8)
	Chickens	227	17			2	1	12	60	133	2			32	32	64	2 (0.9)
	Ducks	155	7			3		6	47	86	2	3	1	32	32	≥ 256	6 (3.9)
	Wild birds	60	26				2	3	15	14				8	32	32	0

### Antimicrobial susceptibility testing

The MICs of fosfomycin (Sigma Chemical Co., St. Louis, Missouri) were determined by the agar plate dilution method. The bacterial strains were initially cultured in 5% sheep blood agar plate (Komed, Seongnam, South Korea) for 48 h under microaerobic conditions (10% CO_2_, 5% O_2_, and 85% N_2_) at 42°C; the culture was then suspended in Mueller–Hinton broth (Oxoid Ltd., Basingstoke, England) to obtain a suspension of 0.5 McFarland turbidity. Fosfomycin concentrations ranged from 0.025 to 256 μg/mL. Two-fold serial dilutions of fosfomycin were prepared, and the resulting solutions were added to Mueller–Hinton agar (Oxoid) supplemented with 5% sheep blood and 25 μg/mL of glucose-6-phosphate. *E*. *coli* ATCC 25922 and *Staphylococcus aureus* ATCC 29213 were used as the quality control strains. Since the MIC breakpoints for fosfomycin against *Campylobacter* were unknown, we used the following breakpoints for *Enterobacteriaceae* from European Committee for Antimicrobial Susceptibility Testing (EUCAST): susceptible, ≤ 32 μg/mL and resistant, ≥ 64 μg/mL [25]. MIC_50_, MIC_90_, and MIC_100_ values were defined as the lowest concentration of the antibiotic at which 50%, 90%, and 100% of the isolates were inhibited, respectively.

### Polymerase chain reaction

We performed partial gene amplification of the *fosX*^*CC*^ gene of chromosomal DNA from *Campylobacter* strains with an MIC ≥ 64 μg/mL. DNA templates were prepared using *Campylobacter* colonies freshly grown on blood agar by adding 100-μl sterile distilled water and boiling in a heater block at 100°C for 15 min. The template DNA was stored at −20°C until it was used for the polymerase chain reaction (PCR). Long, single-stranded oligonucleotides of 426 bp for the whole *fosX*^*CC*^ gene were synthesized based on the reference strain DZB4 (GenBank accession number KC876749) and used as the PCR positive control (Bioneer Inc., Daejeon, South Korea). Oligonucleotide primers for the *fosX*^*CC*^ gene were synthesized using our own design: fosX-F (5′-TCAAAGACCTAGATAAAGCCAC-3′) and fosX-R (5′-TGTTTCCAATGTTCCAGTGT-3′). DNA amplification was performed in a PCR Thermal Cycler Dice with the following parameters: pre-denaturation at 95°C for 1 min; 35 cycles of denaturation at 95°C for 30 s, annealing at 55°C for 30 s, and extension at 72°C for 1 min; and a final extension at 72°C for 5 min.

### Pulsed-field gel electrophoresis

PFGE was conducted according to the CDC standardized procedure for the molecular subtyping of *Campylobacter* using the CHEF Mapper apparatus (Bio-Rad Laboratories). Genomic DNA was digested with *Sma*I, and *Xba*I-digested DNA from *Salmonella Braenderup* H9812 was used as the standard size.

## Results

MIC distribution of fosfomycin for *Campylobacter jejuni* and *C*. *coli* isolates from different sources is presented in Table 1. The MIC_50_, MIC_90_, and MIC_100_ values were 32, 32, and ≥ 256 μg/mL for *C*. *jejuni* and 32, 32, and 128 μg/mL for *C*. *coli*. The MIC_90_ was 32 μg/mL for all the isolates from chicken, duck, and wild birds. The chicken and duck isolates had a higher MIC_50_ of 32 μg/mL for *C*. *jejuni* than the wild bird isolates (MIC_50_, 8 μg/mL); they also had a higher MIC_100_ for both *C*. *jejuni* and *C*. *coli* than wild birds. The prevalence of chicken (7.5%, 17/227) and duck isolates (4.5%, 7/155) with MIC ≤ 0.25 μg/mL was lower (*p* < 0.01) than that of the wild bird isolates (43.3%, 26/60). Using the available *Enterobacteriaceae* breakpoints, eight (1.8%) strains of *Campylobacter*, including five *C*. *jejuni* isolated from ducks and three *C*. *coli* from duck and chickens, showed resistance to fosfomycin with the MIC values ranging from 64 to ≥ 256 μg/mL. Fosfomycin resistance in chickens, ducks, and wild birds was 0.9% (2/227) and 3.9% (6/155), and 0% (0/60), respectively.

The characterization of fosfomycin-resistant *Campylobacter* strains is shown in Table 2. All eight of the fosfomycin-resistant strains were multidrug-resistant. All five isolates of *C*. *jejuni* were from ducks, including one from broiler duck and four from duck meat. The broiler duck isolate was co-resistant to ampicillin, azithromycin, erythromycin, ciprofloxacin, nalidixic acid, and tetracycline. The other four were co-resistant to ampicillin, ciprofloxacin, nalidixic acid, and tetracycline. One *C*. *coli* from a breeder chicken and one from chicken meat showed multidrug resistance to ampicillin, ciprofloxacin, nalidixic acid, and tetracycline. The remaining one, sourced from duck meat, was resistant to ampicillin, azithromycin, erythromycin, ciprofloxacin, nalidixic acid, and tetracycline. The fosfomycin resistance gene *fosX*^*CC*^ was not detected in the eight fosfomycin-resistant strains. The eight PFGE types showed genetic diversity among the eight fosfomycin-resistant strains (data not shown).

**Table 2:** Characterization of fosfomycin-resistant *Campylobacter* strains in this study.

Species	Strain	Source	Year	Resistance pattern^a^	Fosfomycin MIC (μg/mL)	*fosX*^*CC*^	PFGE pattern
***C*. *jejuni***	D12-MR-009-1	Duck (broiler)	2012	Amp/Azi/Ery/Cip/Nal/Tet	64	-^b^	1
	DM13-FI-006-1	Duck (meat)	2013	Amp/Cip/Nal/Tet	64	-	2
	DM13-JW-WS-015	Duck (meat)	2013	Amp/Cip/Nal/Tet	128	-	3
	DM13-FI-SS-017	Duck (meat)	2013	Amp/Cip/Nal/Tet	128	-	4
	DM13-JDW-SS-010	Duck (meat)	2013	Amp/Cip/Nal/Tet	≥256	-	5
***C*. *coli***	A16-CF-130-2-S2	Chicken (breeder)	2016	Amp/Cip/Nal/Tet	64	-	6
	A16-CF-254	Chicken (meat)	2016	Amp/Cip/Nal/Tet	64	-	7
	DM13-FI-WS-015	Duck (meat)	2013	Amp/Azi/Ery/Cip/Nal/Tet	128	-	8

^a^Ampicillin, Amp; azithromycin, Azi; ciprofloxacin, Cip; erythromycin, Ery; nalidixic acid, Nal; tetracycline, Tet.

^b^PCR negative.

## Discussion

This study was to investigate fosfomycin resistance among the *Campylobacter* isolates obtained from chicken, duck, and wild birds; it revealed fosfomycin resistance in chickens and ducks. To our knowledge, this is the first report of a study investigating the fosfomycin resistance of *Campylobacter* isolates in ducks and wild birds. Our results showed that a small fraction (1.8%) of *Campylobacter* isolates from chickens and ducks was resistant to fosfomycin, and no fosfomycin resistance was found in wild birds. This result supports a previous study [17] which reported good efficacy of fosfomycin against *Campylobacter* isolates sourced from chicken.

From our result, all eight isolates resistant to fosfomycin were obtained from chickens and ducks. This result confirmed the previous report that fosfomycin resistance may have existed in domestic animals since quite a long time ago. In addition, Sackey et al. [17] reported no fosfomycin-resistant *Campylobacter* spp. in live chickens in Ghana. Schwaiger et al. [18] studied organically and conventionally kept laying hens in Germany; they confirmed fosfomycin-resistance with an MIC higher than 32 μg/mL in 22.9% and 11.1% of *C*. *jejuni* and 24.0% and 22.2% of *C*. *coli*, respectively. Recently, Wang et al. [19] reported a strain of *C*. *coli* with a high level of (MIC > 512 μg/mL) resistance to fosfomycin from swine in China. Our results showed that the MIC_50_ values of *Campylobacter* isolates were 32 μg/mL in poultry (chickens and ducks) isolates and 8 μg/mL in wild bird isolates. Since there was no record of fosfomycin treatment in poultry in this study, the higher MIC value of *Campylobacter* in poultry than in wild birds may suggest that the environment in the farm may promote the difference in the MIC values [26].

Increasing interest in fosfomycin potency is because fosfomycin has not been reported to have cross-resistance to any other known antibacterial agent, and it has antibacterial activity against many drug-resistant bacteria [27]. Furthermore, fosfomycin was previously recommended to treat macrolide- and fluoroquinolone-resistant *Campylobacter* enteritis, and it showed good activity [7]. The fosfomycin-resistant *Campylobacter* isolates, which were co-resistant to macrolide and fluoroquinolone, have previously been reported in humans. Gomezgarces et al. [20] reported that from among 60 ciprofloxacin-resistant *Campylobacter* strains isolated from acute diarrheal infections, 16.6% had MIC > 128 μg/mL. In addition, Sorlozano-Puerto et al. [28] reported fosfomycin co-resistance to ciprofloxacin and macrolides. In our study, all eight fosfomycin-resistant *Campylobacter* strains were multidrug resistant; six strains were co-resistant to fluoroquinolone, ampicillin, and tetracycline, and two strains (D12-MR-009-1 & DM13-FI-WS-015) were co-resistant to fluoroquinolone, ampicillin, tetracycline, and macrolide (Table 2). Despite the reason for fosfomycin resistance occurrence in food animals, the transmission direction of fosfomycin resistance between humans and these animals remains unknown. Nevertheless, the increasing resistance of *Campylobacter* strains to fosfomycin and other therapeutic antibiotics in such animals indicates a significant public health threat.

In our study, six fosfomycin-resistant *Campylobacter* strains from duck, including one strain from broiler ducks and five strains from duck meat, were identified; this suggests that ducks are a significant channel for fosfomycin resistance transmission. Further attention is warranted on the two strains of fosfomycin-resistant *Campylobacter* that were co-resistant to fluoroquinolone, ampicillin, tetracycline, and macrolide. If fosfomycin resistance is present in ducks, which always feed in open air systems and are sometimes raised under free-range animal husbandry method in Korea, then they may transmit the resistance more quickly and more easily than chicken.

Several fosfomycin resistance mechanisms have been described in gram-negative bacteria, including target modification, expression of antibiotic-degrading enzymes, reduced uptake, and rescue of the UDP-MurNAc biogenesis pathway [27]. In *Campylobacter*, the fosfomycin resistance gene *fosX*^*CC*^ is located in a transferable chromosomal multidrug resistance genomic island that harbors multiple antibiotic resistance determinants (macrolides, aminoglycosides, and tetracycline) [19]. In this study, *fosX*^*CC*^ was not detected in the eight fosfomycin-resistant strains. The precise molecular level understanding of the mechanisms of reduced susceptibility to fosfomycin seen in these strains remains unclear, and further evaluation of the fosfomycin resistance mechanism in *Campylobacter* is required. The target mutation, plasmid-mediated fosfomycin glutathione S-transferase genes could also induce fosfomycin resistance in gram-negative bacteria [27]. Furthermore, it has been reported that efflux pump may play a role in fosfomycin resistance [29]. Thus, further studies investigating fosfomycin resistance mechanism in *Campylobacter* may focus on these aspects.

It is well known that no breakpoint for fosfomycin against *Campylobacter* spp. has been defined by EUCAST, Clinical and Laboratory Standards Institute (CLSI), British Society for Antimicrobial Chemotherapy (BSAC), or any other body. In this study, although we followed the breakpoint defined for *Enterobacteriaceae* and showed eight fosfomycin-resistant strains isolated from poultry, it may not reflect the actual efficacy of fosfomycin against *Campylobacter*. Further studies are required to establish the breakpoint of fosfomycin against *Campylobacter*. The MIC results from numerous *Campylobacter* spp. isolates from poultry and wild birds in this study will benefit this purpose of defining this breakpoint.

In conclusion, this study is particularly significant as the resistance of *Campylobacter* spp. to other antibiotics is increasing, thus compromising the decision of the ideal empirical and definitive treatment. The local susceptibility data should always guide treatment decisions. The reports of the failure of traditional antimicrobial drugs, such as fluoroquinolones and macrolides, are increasing, and thus, fosfomycin may be a valuable treatment option as the last-resort for the treatment of campylobacteriosis. Fosfomycin resistance has been emerging and spreading in food animals; thus, this resistance can also transmit to humans along the food chain. Therefore, to better understand fosfomycin resistance in *Campylobacter* in such animals, the resistance mechanisms and the mode of transmission require deeper study. Furthermore, an appropriate MIC or zone diameter breakpoint for *Campylobacter* needs to be established to define the resistance in the future.

## References

[1] Engberg J, Neimann J, Nielsen EM, Aarestrup FM, Fussing V. Quinolone-resistant *Campylobacter* infections in Denmark: Risk factors and clinical consequences. Emerg Infect Dis. 2004;10(6): 1056–63. 10.3201/eid1006.030669 15207057 PMC3323146

[2] European Food Safety Authority (EFSA). The European Union summary report on antimicrobial resistance in zoonotic and indicator bacteria from humans, animals and food in 2016. EFSA J. 2018;16(2). doi: Unsp 4380 10.2903/J.Efsa.2018.5182 PMC7009656 32625816

[3] Zhou JY, Zhang MJ, Yang WN, Fang YQ, Wang GQ, Hou FQ. A seventeen-year observation of the antimicrobial susceptibility of clinical *Campylobacter jejuni* and the molecular mechanisms of erythromycin-resistant isolates in Beijing, China. Int J Infect Dis. 2016;42: 28–33. 10.1016/j.ijid.2015.11.005 26594011

[4] Kim JS, Lee MY, Kim SJ, Jeon SE, Cha I, Hong S, et al High-level ciprofloxacin-resistant *Campylobacter jejuni* isolates circulating in humans and animals in Incheon, Republic of Korea. Zoonoses Public Health. 2016;63(7): 545–54. 10.1111/zph.12262 27234414

[5] Lehtopolku M, Nakari UM, Kotilainen P, Huovinen P, Siitonen A, Hakanen AJ. Antimicrobial susceptibilities of multidrug-resistant *Campylobacter jejuni* and *C*. *coli* strains: *in vitro* activities of 20 antimicrobial agents. Antimicrob Agents Chemother. 2010;54(3): 1232–6. 10.1128/AAC.00898-09 20038624 PMC2825995

[6] National Antimicrobial Resistance Monitoring System (NARMS). NARMS Retail Meat Annual Report 2011. U.S. Food and Drug Administration; 2011.

[7] Aguilar-Company J, Los-Arcos I, Pigrau C, Rodriguez-Pardo D, Larrosa MN, Rodriguez-Garrido V, et al Potential Use of fosfomycin-tromethamine for treatment of recurrent *Campylobacter* species enteritis. Antimicrob Agents Chemother. 2016;60(7): 4398–400. 10.1128/AAC.00447-16 27161640 PMC4914669

[8] Gaudreau C, Rodrigues-Coutlee S, Pilon PA, Coutlee F, Bekal S. Long-lasting outbreak of erythromycin- and ciprofloxacin-resistant *Campylobacter jejuni* subspecies *jejuni* from 2003 to 2013 in men who have sex with men, Quebec, Canada. Clin Infect Dis. 2015;61(10): 1549–52. 10.1093/cid/civ570 26187024

[9] Shin E, Hong H, Oh Y, Lee Y. First report and molecular characterization of a *Campylobacter jejuni* isolate with extensive drug resistance from a travel-associated human case. Antimicrob Agents Chemother. 2015;59(10): 6670–2. 10.1128/AAC.01395-15 26239988 PMC4576103

[10] Tribble DR. Resistant pathogens as causes of traveller's diarrhea globally and impact(s) on treatment failure and recommendations. J Travel Med. 2017;24: S6–S12. 10.1093/jtm/taw090 28520997 PMC5731445

[11] Xu HW, Qin SS, Liu HM. New synthetic antibiotics for the treatment of *Enterococcus* and *Campylobacter* infection. Curr Top Med Chem. 2014;14(1): 21–39. 24236728 10.2174/1568026613666131113145544

[12] Kurekci C, Padmanabha J, Bishop-Hurley SL, Hassan E, Al Jassim RAM, McSweeney CS. Antimicrobial activity of essential oils and five terpenoid compounds against *Campylobacter jejuni* in pure and mixed culture experiments. Int J Food Microbiol. 2013;166(3): 450–7. 10.1016/j.ijfoodmicro.2013.08.014 24041998

[13] Kurincic M, Klancnik A, Mozina SS. Epigallocatechin gallate as a modulator of *Campylobacter* resistance to macrolide antibiotics. Int J Antimicrob Agents. 2012;40(5): 467–71. 10.1016/j.ijantimicag.2012.07.015 22999765

[14] Van Hoang K, Stern NJ, Saxton AM, Xu FZ, Zeng XM, Lin J. Prevalence, development, and molecular mechanisms of bacteriocin resistance in *Campylobacter*. Appl Environ Microbiol. 2011;77(7): 2309–16. 10.1128/AEM.02094-10 21278269 PMC3067428

[15] Nogawa T, Takeuchi Y, Watanabe J, Kimura K, Tomii I, Abe T, et al [Clinical trial of fosfomycin for *Campylobacter* enteritis]. Jpn J Antibiot. 1984;37(9): 1620–4. 6512981

[16] Fuchigami T, Ootaki A, Fujita Y, Yoza A, Ryo S, Nishiyama N. [Evaluation of fosfomycin in *Campylobacter jejuni* enteritis]. Jpn J Antibiot. 1983;36(10): 2849–55. 6674520

[17] Sackey BA, Mensah P, Collison E, Sakyi-Dawson E. *Campylobacter*, *Salmonella*, *Shigella* and *Escherichia coli* in live and dressed poultry from metropolitan Accra. Int J Food Microbiol. 2001;71(1): 21–8. 11764888 10.1016/s0168-1605(01)00595-5

[18] Schwaiger K, Schmied EMV, Bauer J. Comparative analysis of antibiotic resistance characteristics of Gram-negative bacteria isolated from laying hens and eggs in conventional and organic keeping systems in Bavaria, Germany. Zoonoses Public Health. 2008;55(7): 331–41. 10.1111/j.1863-2378.2008.01151.x 18667026

[19] Wang Y, Yao H, Deng F, Liu D, Zhang Y, Shen Z. Identification of a novel *fosX*^*CC*^ gene conferring fosfomycin resistance in *Campylobacter*. J Antimicrob Chemother. 2015;70(4): 1261–3. 10.1093/jac/dku488 25433007

[20] Gomezgarces JL, Cogollos R, Alos JI. Susceptibilities of fluoroquinolone-resistant strains of *Campylobacter jejuni* to 11 oral antimicrobial agents. Antimicrob Agents Chemother. 1995;39(2): 542–4. 10.1128/Aac.39.2.542 7726529 PMC162576

[21] Osterblad M, Norrdahl K, Korpimaki E, Huovinen P. Antibiotic resistance—How wild are wild mammals? Nature. 2001;409(6816): 37–8. 10.1038/35051173 11343104

[22] Wei B, Cha SY, Yoon RH, Kang M, Roh JH, Seo HS, et al Prevalence and antimicrobial resistance of *Campylobacter* spp. isolated from retail chicken and duck meat in South Korea. Food Control. 2016;62: 63–8. 10.1016/j.foodcont.2015.10.013

[23] Wei B, Cha SY, Kang M, Roh JH, Seo HS, Yoon RH, et al Antimicrobial susceptibility profiles and molecular typing of *Campylobacter jejuni* and *Campylobacter coli* isolates from ducks in South Korea. Appl Environ Microbiol. 2014;80(24): 7604–10. 10.1128/AEM.02469-14 25261524 PMC4249223

[24] Wei B, Cha SY, Kang M, Jang HK. Dissemination of multidrug-resistant *Campylobacter* in wild birds from South Korea. Int J Antimicrob Agents. 2015;45(2): 197–8. 10.1016/j.ijantimicag.2014.10.007 Epub 2014 Nov 4. 25468716

[25] ECUAST. Breakpoint tables for interpretation of MICs and zone diameters, Version 7.12017. Available from: http://www.eucast.org/fileadmin/src/media/PDFs/EUCAST_files/Breakpoint_tables/v_7.1_Breakpoint_Tables.pdf

[26] McMahon MAS, Xu JR, Moore JE, Blair IS, McDowell DA. Environmental stress and antibiotic resistance in food-related pathogens. Appl Environ Microbiol. 2007;73(1): 211–7. 10.1128/AEM.00578-06 17142359 PMC1797128

[27] Silver LL. Fosfomycin: Mechanism and resistance. Csh Perspect Med. 2017;7(2). doi: ARTN a025262 10.1101/cshperspect.a025262 28062557 PMC5287057

[28] Sorlozano-Puerto A, Navarro-Mari JM, Gutierrez-Fernandez J. Activity of fosfomycin on clinical isolates of *Campylobacter jejuni* and *Campylobacter coli* of enteric origin. Antimicrob Agents Chemother. 2017;61(2). doi: ARTN e02317-16 10.1128/AAC.02317-16 28119317 PMC5278721

[29] Sharma A, Sharma R, Bhattacharyya T, Bhando T, Pathania R. Fosfomycin resistance in *Acinetobacter baumannii* is mediated by efflux through a major facilitator superfamily (MFS) transporter-AbaF. J Antimicrob Chemother. 2017;72(1): 68–74. 10.1093/jac/dkw382 27650185

